# Intralesional Marginal Resection for Osteoblastoma in the Mobile Spine: Experience From a Single Center

**DOI:** 10.3389/fsurg.2022.838235

**Published:** 2022-06-06

**Authors:** Shiliang Cao, Keyuan Chen, Liang Jiang, Feng Wei, Xiaoguang Liu, Zhongjun Liu

**Affiliations:** Department of Orthopaedics, Peking University Third Hospital, Beijing, China

**Keywords:** osteoblastoma, enneking system, spine, resection, treatment

## Abstract

Osteoblastoma (OB) is a benign bone tumor with aggressive behavior and a tendency for local recurrence. The appropriate surgical strategy for spinal OB remains unclear. This retrospective study aimed to verify the clinical efficacy and safety of intralesional marginal resection of OB in the mobile spine. We enrolled 50 consecutive patients with spinal OB between January 2009 and December 2019. The tumors were staged based on the Enneking system, with 21 and 29 lesions being determined as stage 2 (St.2) and stage 3 (St.3), respectively. Among them, 42 patients underwent intralesional marginal resection, five underwent extensive curettage, and three underwent en bloc resection successfully since their lesions were limited to the posterior element in a single vertebra. We analyzed clinical characteristics, perioperative and follow-up images, surgical details, and follow-up data. Within a median follow-up duration of 50 (range: 24–160) months, six (12.0%) patients had local recurrence. The recurrence rates among patients who underwent intralesional marginal resection, curettage, en bloc resection were 7.1%(3/42), 60.0%(3/5), and 0%(0/3), respectively. The recurrence rate of intralesional marginal resection of St.3 lesions was slightly higher than that of St.2 lesions (7.7%[2/26] vs. 6.3%[1/16]). There were 16(38.1%), 3(60.0%), and 0 patients with surgical complications among those who underwent intralesional marginal resection, curettage, and en bloc resection, respectively. Local recurrence was observed in five (5/14, 35.7%) patients who had vertebral artery extension and in none who did not have vertebral artery extension (*p *= 0.02). Our findings suggest that intralesional marginal resection could be an appropriate treatment choice for patients with spinal OB, both St.2 and St.3 lesions, with an acceptable local recurrence rate and a low risk of complications. Vertebral artery extension could be a strong risk factor for local recurrence in patients with spinal OB.

## Introduction

Osteoblastoma (OB) accounts for approximately 1% of primary bone tumors (1–3), and the majority occur in the spine (32%–46%) (1–6). Spinal OB accounts for 10% of all spinal bone tumors and tends to invade the posterior spinal elements (1, 2, 6). Enneking Stage 3 (St.3) spinal OB presents an increasingly aggressive and expansive behavior, causes bone destruction and soft tissue extension, and has a tendency for local recurrence (7). They can also cause pathological fractures and neurological impairment and even undergo malignant transformation after repeated surgical interventions (6–8).

Surgical resection remains the primary curative modality for spinal OB (2, 6, 8–10); however, the optimal surgical strategy remains unclear. En bloc resection is recommended for spinal OB to minimize the local recurrence rate, especially for St.3 lesions. Some studies have been conducted on the efficacy and safety of en bloc resection for spinal OB (11–15); however, given the complex anatomical structure of the spine, en bloc resection is technically challenging, which leads to a relatively high surgical complication rate (12, 13, 16).

Since spinal OB is not a malignant tumor, and its recurrence rate is relatively low, furthermore, intralesional curettage remains the standard surgery for OB located in the extremities (17, 18). Some studies have suggested that intralesional resection and en bloc resection have similar clinical efficacies and local recurrence rates in patients with spinal OB (9, 19, 20). Therefore, we speculated that intralesional marginal resection could be an appropriate choice for spinal OB. This retrospective study aimed to evaluate the local recurrence and clinical efficacy of intralesional marginal resection in patients with spinal OB.

## Methods

### General Information

This study was approved by our hospital’s ethics committee and was conducted following the principles of the Declaration of Helsinki. The inclusion criteria were as follows: postoperative pathological diagnosis of spinal OB, surgical treatment, and a minimum follow-up period of 24 months. A review of our spinal tumor database identified 51 patients with spinal OB who had undergone surgical treatment at our hospital between January 2009 and December 2019.

We retrospectively evaluated prospectively recorded hospital charts, operating room reports, anesthesia reports, office charts, pathology reports, and radiographs. We collected the following data: age, sex, symptoms, underlying diseases, smoking habits, body mass index, neurologic function, Enneking stage, surgical procedure, pathology, and treatment complications. Neurological function was evaluated using the modified Frankel grade at admission and final follow-up.

Enneking staging was performed based on the radiological findings (21). Enneking Stage 2 (St.2, active lesion) was considered as an osteolytic lesion surrounded by osteosclerosis with well-defined borders, with varying degrees of ossification or calcification in the lesion. Enneking Stage 3 (St.3, aggressive lesion) referred to the expansile osteolytic lesion with notable cortical bone destruction, often accompanied by paravertebral or intraspinal soft tissue masses and indistinct lesion margins (22). Additionally, we documented the extension of OB lesions to the vertebral artery.

### Imaging and Biopsy

All patients routinely underwent posteroanterior and lateral spinal radiography, computed tomography (CT), and magnetic resonance imaging (MRI). For patients with atypical images of the spinal OB, interventional radiologists performed a CT-guided biopsy.

### Follow-up

After the index procedure, roentgenograms were performed at the 3- and 6-month follow-up visits, as well as at 6-month intervals within the next 2 years and annually thereafter. CT and MRI were performed at the 3-month follow-up and annually thereafter. Immediate CT and MRI were performed when a patient showed symptoms indicative of local recurrence. If necessary, positron emission tomography-CT (PET-CT) and isotope bone scans were prescribed for suspected local recurrence.

### Surgical Techniques

En bloc resection can be easily performed for small lesions of spinal OB, especially in the lamina. In our institution, intralesional marginal resection is usually performed and begins with careful exposure of the lesion through the normal tissue. To protect crucial surrounding structures (e.g., the vertebral artery), the tumor capsule could be violated, and the tumor removed in two or more pieces. If possible, preoperative selective artery embolism was routinely performed 24 h prior to surgery to minimize intraoperative blood loss. Radiotherapy was only recommended for patients with local recurrence but difficult for reoperation.

### Statistical Analysis

Clinical data are presented as the mean and standard deviation (SD) for continuous variables and as frequency counts and percentages for discrete variables. Fisher’s exact test was used to analyze categorical variables based on the sample size and expected value. Statistical significance was set at *p* < 0.05. Statistical analysis was performed using SPSS (version 20.0; SPSS, Inc., Chicago, IL).

## Results

We included 51 consecutive patients with spinal OB; among them, 50(98.0%) patients had a median follow-up period of 50 months (range: 24–160 months) (Table 1). Among these 50 patients, there were 34(68.0%) males and 16(32.0%) females (mean age during operation: 25.8 ± 13.9 [7–58] years). The lesions were located in the cervical, thoracic, and lumbar spine in 29, 14, and 7 patients, respectively. The lesions were St.2 and St.3 in 21(42.0%) and 29(58.0%) patients, respectively. Five patients were referred to us after previous unsuccessful surgeries.

**Table 1:** Demographic and clinical characteristics of the patients.

Characteristic	Value
Case number (*n*)	50
Mean age at operation^a^ (years)	25.8 ± 13.9
Gender (*n*,% Male)	34(68.0%)
Symptoms	
Localized pain (*n*,%)	47(94.0%)
Night pain (*n*,%)	20(39.2%)
Extremity weakness (*n*,%)	8(15.7%)
Radicular pain (*n*,%)	7(13.7%)
Frankel Classification	
Frankel Grade A-D	8(16.0%)
Frankel Grade E	42(84.0%)
Spinal Location	
Cervical (*n*,%)	29(58.0%)
Thoracic (*n*,%)	14(28.0%)
Lumbar (*n*,%)	7(14.0%)
Enneking Classification	
S2 Stage	21(42.0%)
S3 Stage	29(58.0%)
Previous spine tumor operation (*n*,%)^b^	5(10.0%)
Vertebral Artery extension (*n*,%)	14(28.0%)
Secondary Aneurysmal Bone Cyst (*n*,%)	6(12.0%)
Follow-up month (median, range)	50(24–160)

^a^ *The values are given as the mean and the standard deviation.*

^b^ *These patients were defined as non-intact patients.*

In our study, 42 patients underwent intralesional marginal resection, while 5 patients underwent extensive curettage due to excessive intraoperative bleeding. Three patients successfully underwent en bloc resection since their lesions were St.2 and limited to the posterior element (lamina or transverse process) in a single vertebra, which could be easily removed through a single posterior approach.

At the final follow-up, six (12%) patients had local recurrence, including three patients each who underwent intralesional marginal resection (7.1%) and curettage (60.0%). The median time interval from surgery to recurrence was 11 months (range: 7–41 months). Further, the total local recurrence rates for St.2 and St.3 lesions were 9.5% (2/21) and 13.8% (4/29), respectively. Among patients who underwent intralesional marginal resection, the recurrence rates for St.2 and St.3 lesions were 6.3% (1/16) and 7.7% (2/26), respectively (Table 2).

**Table 2:** Local recurrence rate.

	Total	Intralesional marginal resection	En bloc resection	Curettage
Enneking S2 Stage	21	16	3	2
Recurrence	2(9.5%)	1(6.3%)	0(0%)	1(50.0%)
Enneking S3 Stage	29	26	0	3
Recurrence	4(13.8%)	2(7.7%)	0(0%)	2(66.7%)
Total	50	42	3	5
Recurrence	6(12.0%)	3(7.1%)	0(0%)	3(60.0%)

*Discrete variables were presented as frequency counts and percentages(n, %) in table*

**Figure 1:**
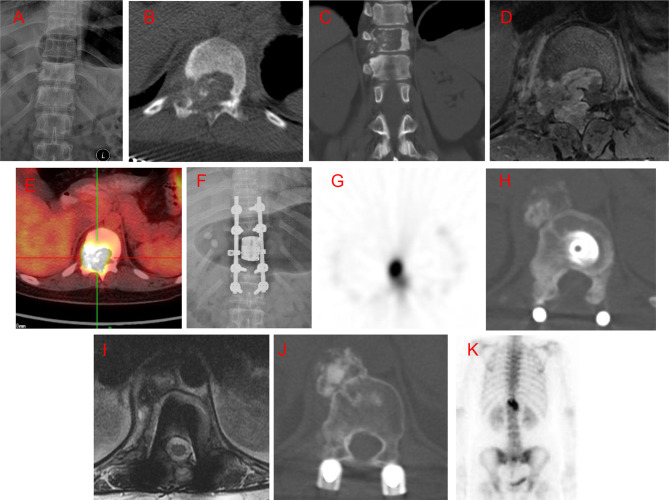
Case 4. A 28-year-old female patient with low back pain for 6 months, with rapid symptom progression to paralysis of the lower extremities within 2 weeks. An osteolytic lesion was located in the T11 vertebral body, right pedicle, transverse process, and lamina. She underwent an intralesional vertebrectomy; however, computed tomography (CT) and isotope bone scans at 20 months postoperative revealed a local recurrence. She underwent radiotherapy but refused advanced surgery. The recurrent lesion was stable and gradually ossified, as seen at the 16-month follow-up after radiotherapy. (**A**) Preoperative posteroanterior radiography. (**B,C**) Preoperative axial and coronal CT scans. (**D**) T2-weighted magnetic resonance imaging (MRI) scan. (**E**) Preoperative positron emission tomography-CT (PET-CT). (**F**) Posteroanterior radiography 1 week postoperatively. (**G**) Isotope bone scan 20 months postoperatively. (**H**) Axial CT scan 20 months postoperatively. (**I**) T2-weighted MRI scan 20 months postoperatively. (**J**) Axial CT scan 16 months after radiotherapy. (**K**) Isotope bone scan 16 months after radiotherapy.

**Figure 2:**
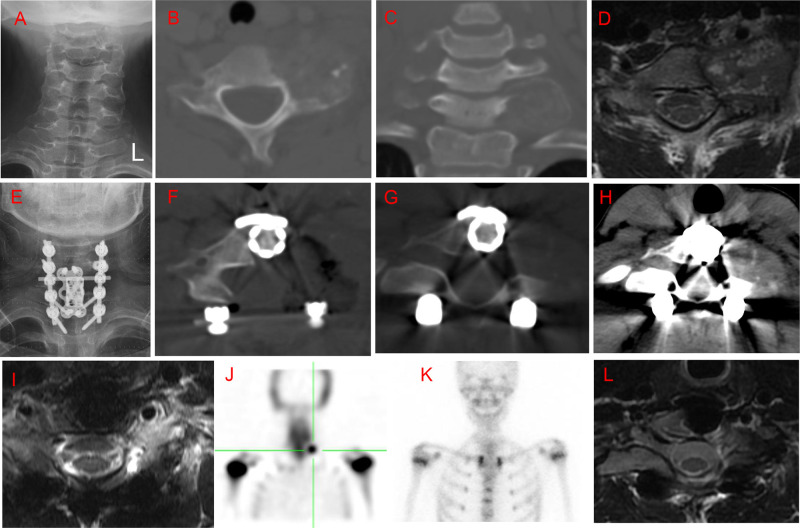
Case 5. A 15-year-old male patient with local neck and night pain for 6 months. An osteolytic lesion was located in the C7 vertebral body, left pedicle, transverse process, and lateral mass, with left vertebral artery extension. He underwent intralesional marginal resection; however, positron emission tomography-computed tomography (PET-CT) 7 months postoperatively showed local recurrence. The patient underwent a second surgery through a posterior approach. There was no further recurrence at a 5-year follow up after the second surgery. (**A**) Preoperative posteroanterior radiography. (**B,C**) Preoperative axial and coronal computed tomography (CT) scans. (**D)**: T2-weighted magnetic resonance imaging (MRI) scan. (**E**) Posteroanterior radiography 1 week after the first surgery. (**F**). Axial CT scan 1 week after surgery. (**G,H**) Axial CT scan 7 months after surgery. (**I**) T2-weighted MRI scan 7 months after surgery. (**J**) Isotope bone scan 7 months after surgery. (**K**) Isotope bone scan 3 years after the second surgery. (**L**) T2-weighted MRI scan 5 years after the second surgery.

**Figure 3:**
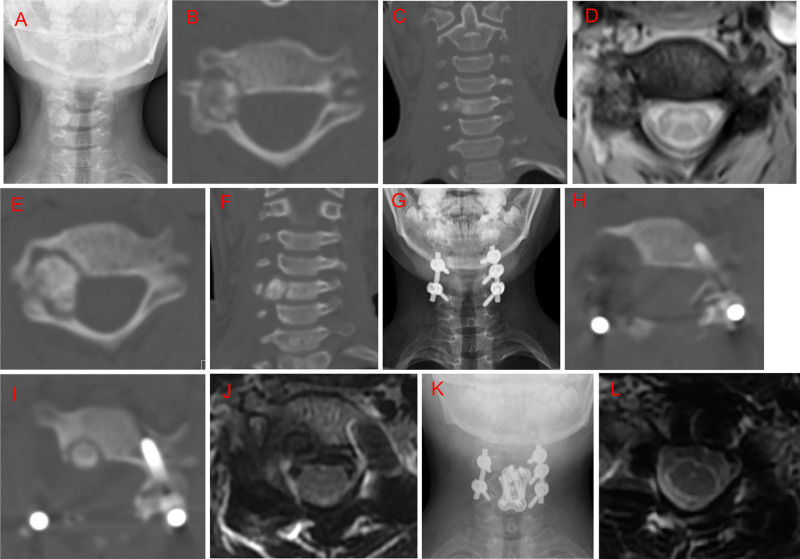
Case 6. A 10-year-old female patient with a history of neck pain over 6 months. An irregular and heterogeneous lesion was located in the C5 right pedicel and transverse process, vertebral body, and lamina, with right vertebral artery extension. She initially underwent radiofrequency ablation; however, computed tomography (CT) examination at the 5-month follow-up revealed lesion enlargement. Subsequently, the patient underwent intralesional marginal resection through a posterior approach. Local recurrence was identified by CT at the 12-month follow-up. She underwent a C5 vertebrectomy through an anterior approach. No recurrence was observed at the 21-month follow up after the second surgery. (**A**) Posteroanterior radiography before radiofrequency ablation. (**B,C**) Preoperative axial and coronal CT scans before radiofrequency ablation. (**D**) T2-weighted magnetic resonance imaging (MRI) scan at 5 months after radiofrequency ablation. (**E,F**) Axial and coronary CT scan at 5 months after radiofrequency ablation. (**G**) Posteroanterior radiography 1 week postoperatively. (**H**) Axial CT scan 1 week postoperatively. (**I**) Axial CT scan 12 months postoperatively. (**J**) T2-weighted MRI scan 12 months postoperatively. (**K**) Posteroanterior radiography 1 week after the second surgery. (**L**) T2-weighted MRI CT scan 21 months after the second surgery.

Of the patients who showed recurrence, one patient (Case 1, St.3, curettage) died of tracheal and esophageal compression after three repeat surgeries and radiotherapy. Among the three patients who survived with the disease at the last follow-up, one patient underwent secondary curettage, and postoperative radiotherapy (Case 2, St.3, curettage), one (Case 3, St.2, curettage) refused revision surgery or radiotherapy, and the other (Case 4, St.3, intralesional marginal resection) (Figure 1) only received radiotherapy after local recurrence. Two patients lacked evidence of disease at the last follow-up after secondary marginal intralesional resection (Case 5, St.3, intralesional marginal resection (Figure 2); Case 6, St.2, intralesional marginal resection (Figure 3)) (Tables 3, 4).

**Table 3:** Study characteristics of recurrence cases.

No.	Gender	Age	Prior treatment	Location	Frankel scale	Surgery	No recurrence months	Last follow-up months	Last status
1	M	48	No	C2-3	E	Curettage	24	103	DOD^a^
2	M	16	No	C2	E	Curettage	41	148	SWD
3	F	24	No	C5	E	Curettage	8	24	SWD
4	F	28	No	T11	D	IMR	20	36	SWD
5	M	15	No	C7	E	IMR	7	65	NED
6	F	10	Ra	C5	E	IMR	12	33	NED

*M, male; F, female; RA, radiofrequency ablation; IMR, intralesional marginal resection; DOD, dead of disease; NED, no evidence of disease; SWD, survival with disease.*

^a^ *Case 1 died of the trachea and esophagus compression after three surgeries and radiotherapy.*

**Table 4:** Imaging characteristics of recurrence cases.

No.	Location	WBB	Enneking stage	Multisegment involvement	C1/2 invasion	Vertebral artery extension	Fluid-fluid levels
1	NA	3–5, A-B	3	Yes	Yes	Yes	No
2	VB + NA	4–11, B-D	3	No	Yes	Yes	No
3	VB + NA	8–10, B-C	2	No	No	Yes	No
4	VB + NA	1–4, B-D	3	No	No	–	Yes
5	VB + NA	3–5, A-B	3	No	No	Yes	No
6	VB + NA	8–11, B-C	2	No	No	Yes	No

*NA, neural arch; VB, vertebral body.*

The location of the spinal OB affected local recurrence. The recurrence rates for cervical, thoracic, and lumbar lesions were 17.2%(5/29), 7.1%(1/14), and 0%(0/7), respectively. The recurrence rates in patients with and without vertebral artery invasion were 35.7% (5/14) and 0%, respectively (*p *= 0.02, Table 5).

**Table 5:** Vertebral artery extension of recurrence cases.

	Total	Recurrence	No recurrence	*p*-value*
Vertebral Artery Extension (*n*,%)	14	5(35.7%)	9(64.3%)	0.02
No Vertebral Artery Extension (*n*,%)	15	0(0%)	15(100%)	

*Fisher's precise test.

Nineteen (38.0%) patients had surgical complications. Among the 42 patients who underwent intralesional marginal resection, there were 16 (38.1%) surgical complications, including six major complications (three cases of pneumonia, two of internal fixation failure, and one of respiratory failure requiring intubation) and 10 minor complications (three cases of wound infections, two of transient hypoxia, two of transient neurological deficits, two of cerebrospinal fluid leakage, and one of urinary tract infection). Among the curettage cases, there were three (60.0%) major surgical complications, including one death (Case 1), one intraoperative vertebral artery injury, and one C5 palsy. There were no complications among the en bloc cases. None of these complications required surgical interventions, except for the two patients with internal fixation failure. At the final follow-up, there were no cases of disability due to surgical complications.

## Discussion

This single-center retrospective study analyzed the surgical outcomes, prognosis, and clinical characteristics of patients with spinal OB. There were 21 and 29 St.2 and St.3 lesions, respectively, with a minimum follow-up duration of 24 months. Among them, 16 St.2 and 26 St.3 lesions were treated with intralesional marginal resection and showed good clinical outcomes. The overall recurrence rate with intralesional marginal resection was 7.1%; the recurrence rate of intralesional marginal resection of St.3 lesions (7.7%) was slightly higher than that of St.2 lesions (6.3%).

Since spinal OB is relatively rare, with only a few large case series being reported, the surgical treatment method remains controversial. Most studies have recommended extended intralesional curettage or marginal resection to completely remove St.2 lesions (8, 23–25). However, the effect of intralesional marginal resection of St.3 lesions remains unclear since St.3 lesions are more aggressive and tend to local recurrence and even malignant transformation after repeated recurrence (7, 8, 19, 21, 24, 25).

According to the Enneking principle, intralesional resection is adequate for benign tumors in musculoskeletal sites on most occasions (14). In the largest review of OB cases from the Mayo Clinic, the local recurrence rate of intralesional resection was 19%, and en bloc resection was 20% in 75 patients who had complete follow-up data (1). Golant et al. proposed that extended intralesional curettage was sufficient for OB in most locations and stated that resection with wide margins is preferred for lesions in some less essential bones (ribs, fibula) (17).

In a study by Boriani et al., 32 and 13 patients with spinal OB underwent intralesional excision and en bloc resection, with recurrence rates of 15.6% (5/32) and 15.4% (2/13), respectively, in the medium-to-long-term follow-up. Further, 22 and 13 patients with St.3 lesions underwent intralesional excision and en bloc excision, with recurrence rates of 23% (5/22) and 15.4% (2/13), respectively (8). Accordingly, they recommended en bloc excision for St.3 spinal OB lesions. In our study, the recurrence rate with intralesional excision of St.3 lesions was 7.7%, which is acceptable.

A multicenter study conducted by Versteeg et al. reported that in 66 patients with spinal OB treated with surgery, the total recurrence rate was 18% (13/73); moreover, the recurrence rates for intralesional resection and en bloc resection were 19% (8/41) and 16% (4/25), respectively (24). This indicates that the recurrence rates for intralesional resection and en bloc resection are similar for spinal OB.

Boriani et al. reported on 50 spinal OB patients who underwent intralesional resection or en bloc resection, including 40 intact lesions and 10 non-intact lesions. In this study, the recurrence rates of en bloc resection for intact and non-intact lesions were 0% (0/10) and 66.7% (2/3), respectively, and the recurrence rates of intralesional resection for intact and non-intact lesions were 6.7% (2/30) and 42.9% (3/7), respectively. Therefore, they recommended en bloc resection for intact spinal OB lesions rather than intralesional resection (8).

In our study, there were five patients with non-intact lesions, and all of them underwent intralesional marginal resection without recurrence at the last follow-up, which suggested that intralesional marginal resection could be appropriate for non-intact lesions with an acceptable local recurrence rate. Moreover, the local recurrence rate of intact patients who received intralesional marginal resection was 5.3% (2/38) in our study, which was acceptable. Therefore, the intact or non-intact factor had no impact on the local recurrence rate in spinal OB patients who received intralesional marginal resection.

Our recurrence rate of intralesional marginal resection overall in spinal OB patients or specifically in St.3 spinal OB patients was parallel with what has been previously reported (6, 7, 8, 19, 24, 25). Therefore, we suggested that intralesional marginal resection could be an appropriate choice for spinal OB, both St.2 and St.3 lesions. Some case reports regarding St.3 spinal OB patients who received intralesional marginal resection had no evidence of disease in 1–4 years follow-up (26–29).

Some studies have recommended en bloc resection for patients with spinal OB to minimize local recurrence; however, it is technically demanding and has a high complication rate (9, 10, 15, 23). Boriani et al. reported 105 (47.7%) major complications, 48(21.8%) minor complications, and 7(3.2%) deaths in 220 patients who underwent en bloc resection for spinal tumors (30). A systematic review of 89 studies reported surgical complication and mortality rates of 13–56% and 0–7.7% for en bloc resection of primary spine tumors, respectively (31). In our study, there were six major (6/42, 14.3%) and ten minor (10/42, 23.8%) complications among patients who underwent intralesional marginal resection, with no deaths or disabilities caused by surgical complications. This indicates that intralesional marginal resection could be safer and less technically demanding than en bloc resection.

Vertebral artery extension can increase the difficulty and complication risk of spinal tumor surgery; however, it is important to preserve and protect the vertebral artery (32). En bloc resection of spinal tumors with vertebral artery extension is challenging, with some reports recommending intralesional marginal resection (26, 28, 33). In our study, all five patients with cervical OB (Case 1, 2, 3, 5, 6) who showed local recurrence had vertebral artery extension. In all patients with vertebral artery extension, the vertebral artery was preserved during surgery; further, the recurrence rate was 35.7% (5/14), which was significantly higher than the overall recurrence rate. Vertebral artery extension increases the possibility of leaving behind residual tumor and increases surgical difficulty due to intraoperative bleeding, which can influence intraoperative visibility. Our finding suggests that vertebral artery extension could be a strong risk factor for local recurrence of spinal OB; this finding needs further validation in prospective, multicenter studies.

This study had several limitations. First, this was a retrospective single-center study, which has an inherent drawback of selection bias. Moreover, only six patients showed local recurrence due to the small sample size. There is a need for prospective, multicenter studies with longer follow-up periods on the treatment of spinal OB.

In conclusion, intralesional marginal resection showed a low local recurrence rate and risk of complications in patients with spinal OB, regardless of lesion stage. The local recurrence rate was similar to those previously reported. Therefore, intralesional marginal resection may be an appropriate choice for patients with spinal OB. Moreover, vertebral artery involvement could be a risk factor for local recurrence in patients with spinal OB.

## Data Availability

The raw data supporting the conclusions of this article will be made available by the authors, without undue reservation.
